# Urban cycling-specific active transportation behaviour is sensitive to the fresh start effect: triangulating observational evidence from real world data

**DOI:** 10.1186/s12966-025-01785-w

**Published:** 2025-06-19

**Authors:** Isaak Fast, Shamsia Sobhan, Nika Klaprat, Tyler George, Nils Vik, Dan Prowse, Jacqueline Collett, Jonathan McGavock

**Affiliations:** 1 Department of Pediatrics, Children’s Hospital Research Institute of Manitoba, University of Manitoba, Winnipeg, Canada; 2 Diabetes Research Envisioned and Accomplished in Manitoba (DREAM Theme), Winnipeg, Canada; 3 Public Works Department, Transportation Division, City of Winnipeg, Winnipeg, Canada; 4 Parlour Coffee, Winnipeg, Canada; 5 Hydro Manitoba Active Transportation Working Group, Hydro Manitoba Inc., Winnipeg, Canada

**Keywords:** Behavioural economics, Heuristics, Physical activity, Cycling, Commuting

## Abstract

**Background:**

This study determined if cycling-specific active transportation (AT) was sensitive to the behavioural economics heuristic “The Fresh Start Effect”, with the beginning of a work week being temporal landmark for cycling to work.

**Methods:**

We triangulated data from five sources to test the study hypothesis. First, publicly available cycling traffic data collected from May to September between 2014 and 2019 using electromagnetic counters (EcoCounter Inc, Montreal Qc.) were used to categorize 5 urban trails as “AT” or “leisure” based on hourly cycling traffic patterns. Linear regression model with repeated measures, compared daily trends in cycling traffic over the course of a work week along the different trail types and then compared with daily trends in occupational bicycle parking (*n* = 56,307 counts), vehicular traffic (*n* = 6.2 M counts), and sales from a local coffee shop (*n* = 166,753 counts) over the same time frame. Effect sizes were compared to daily trends in fitness centre attendance (*n* = 563,290 counts), a positive control for the Fresh Start Effect.

**Results:**

We found a significant ~ 22% decline in daily cycling traffic on both AT (-147 cyclists/day; 95% CI: -199.0 to -95 cyclists/day) and leisure trails (-22 cyclists/day; 95% CI: -59 to + 15 cyclists/day) over the course of a work week. The relative decline over the work week in AT-based cycling traffic was similar to the decline in daily parking (~ 14%; -12 cyclists/day; 95% CI: -17 to -7 cyclists/day). The relative effect size of this trend was nearly identical to the decline in fitness centre attendance over the work week (~ 21%; -592 visits/day; 95% CI: -759 visits/day to -425 visits/day), replicating the original Fresh Start Effect. In contrast to the decline in AT-based cycling traffic, daily vehicular traffic (+ 2248 cars/day; 95% CI: 2022 to + 3674 cars/day) and coffee sales (+ 31 units/day; 95% CI: +22 to + 42 units/day) increased ~ 7% from the beginning to the end of a work week.

**Conclusions:**

The weekly patterns of AT-based cycling are sensitive to the Fresh Start Effect. This observation could be used to inform policies for increasing cycling rates in urban centres.

**Supplementary Information:**

The online version contains supplementary material available at 10.1186/s12966-025-01785-w.

## Background

Populations that engage in regular physical activity experience lower rates of non-communicable diseases compared to populations that do not [1, 2]. Promoting cycling-specific active transportation (AT) is a common public health strategy for increasing population-level physical activity in urban areas [3, 4]. Municipal governments in most high-income countries are investing millions of dollars annually to remodel the built environment to support AT, particularly by creating protected spaces for cycling [5–7]. There is growing evidence that this infrastructure [8]and subsequent use for AT [9] is associated with reduced rates of various chronic diseases in neighbourhoods where they are constructed. Despite the rapid growth in urban cycling infrastructure over the past decade, the primary determinants population-level AT-based cycling behaviour are poorly understood.

**Fig. 1:**
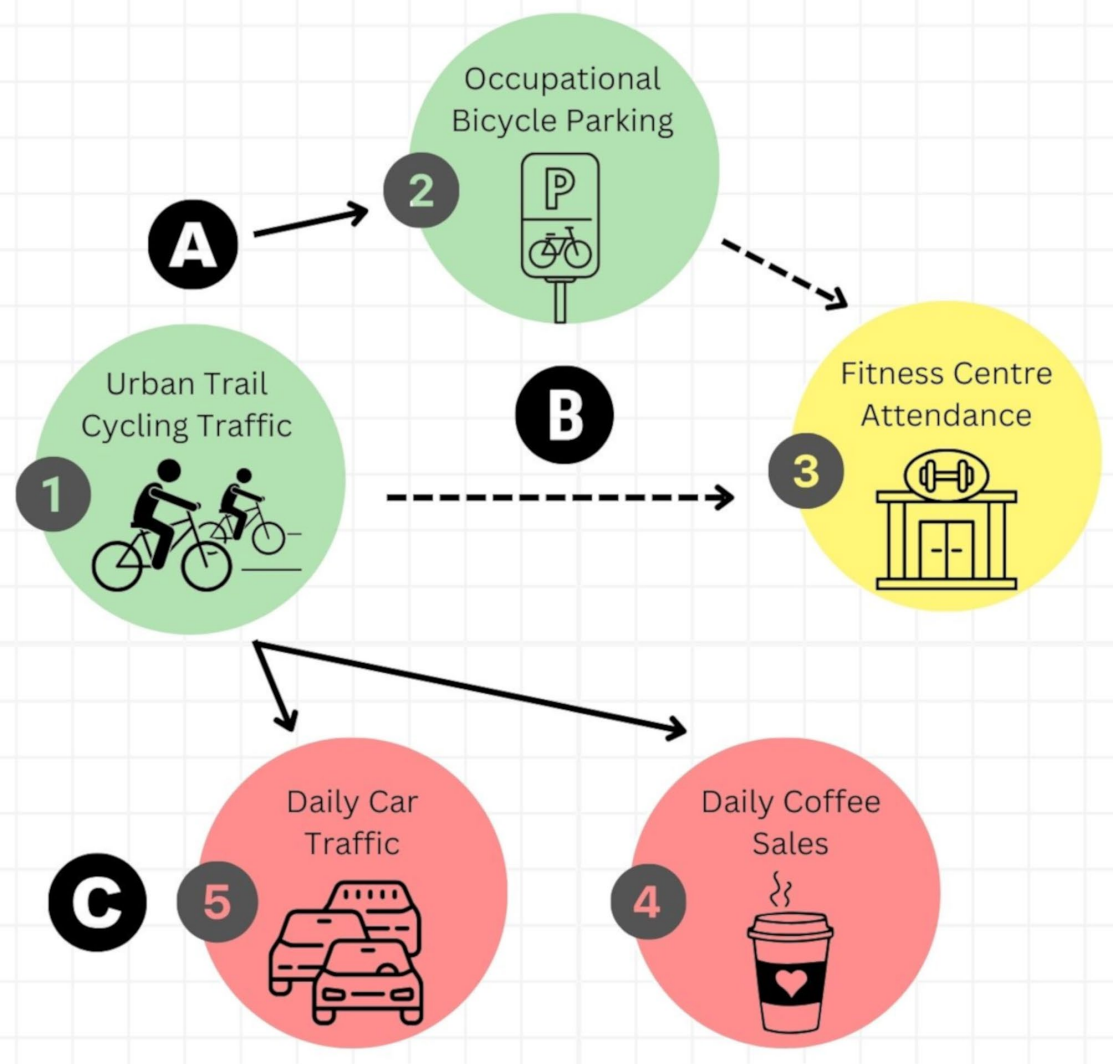
Framework for triangulation of datasets to test the study hypothesis. **(A)** **Main Hypothesis**: Data for population-based cycling active transportation was obtained from (1) multi-use trails from 2014-2019 and (2) a protected bicycle parking space at one of the large employers of the city. **(B)** **Calibration**: Effect sizes for the week patterns of active transportation were compared to weekly trends in (3) fitness centre attendance as this was one of the original objective measures of the Fresh Start Effect related to a physical activity behaviour **(C)** **Negative Controls**: Data used to estimate daily occupational attendance were obtained for (4) a local coffee parlour in the cental business district and (5) vehicular traffic along one of the busiest roads within 5 km of the main university campus and city centre.

Behavioural economics is an area of behavioural science that describes how individual behaviours are governed by heuristics [10]. Several behavioural economic heuristics govern lifestyle behaviours, and are being leveraged to support behaviour change of entire populations [11]. One of these heuristics, The Fresh Start Effect, describes the trend of adopting a new behaviour during a temporal landmark [12]. For example, individuals are more likely to engage in physical activity behaviours at the beginning of a calendar year (New Year’s Resolutions) [13, 14]academic semester [12], birthday, or the beginning of a week [15] compared to days without a distinct temporal landmark. In each case, the modifiable lifestyle behaviours, like leisure physical activity, decline progressively thereafter (i.e. by the end of the month or week). While the Fresh Start effect appears to govern leisure-type physical activity behaviours, it is unclear if it also governs AT-based cycling behaviours. We triangulated real-world data from five different urban contexts (Fig. 1) to determine if urban AT-based cycling behaviours follow a distinct Fresh Start Effect behavioural pattern, characterized by peak traffic at the beginning of a work week and declining thereafter [12]. The primary hypothesis was that cycling traffic along trails characterized by a distinct AT pattern would not exhibit a “Fresh Start Effect”, compared to cycling traffic along trails characterized by leisure-type patterns of use.

## Methods

### Aims, design and setting

We applied the concept of epidemiological triangulation [16] to test the study hypothesis that AT-based cycling does not follow the Fresh Start Effect behavioural heuristic. Specifically, we compared and contrasted weekly trends in (A) leisure-based and AT-based cycling patterns along urban trails; (B) daily rates of use of a protected bicycle parking facility at one of the city’s largest public employers, (C) weekly visits to a local fitness centre; (D) daily vehicle counts along one of the city’s busiest road ways; and (E) coffee purchases at a local coffee shop (Fig. 1). Three datasets were selected specifically to capture and replicate weekly patterns of both AT and leisure-time cycling traffic and leisure physical activity. The fourth (coffee sales) and fifth datasets (vehicular traffic counts) were obtained as negative controls, to estimate trends in working from home and a shift towards non-active transportation. All datasets were collected within the City of Winnipeg, Canada’s 6th largest city, with a population of ~ 841,000.

We secured data that included 1.22 M cycling counts collected over 634 days within 126 weeks along urban trails (2014–2019), 87,794 counts of bicycle parking obtained from 2602 days, within 411 weeks from a large public corporation (2012–2019); 1.95 M individual visits over 839 days within 123 weeks from the local University-based fitness centres (2017–2019), 6,193,449 vehicle counts from 153 days within 22 weeks along one of the city’s busiest roadways (May-Sept 2019) and finally, 366,000 individual coffee sales, from 1504 days, within 313 weeks from the local coffee shop (2012–2019).

#### Research Question #1: Is the Fresh start effect evident for weekly patterns of AT-based cycling behaviours?

**Fig. 2:**
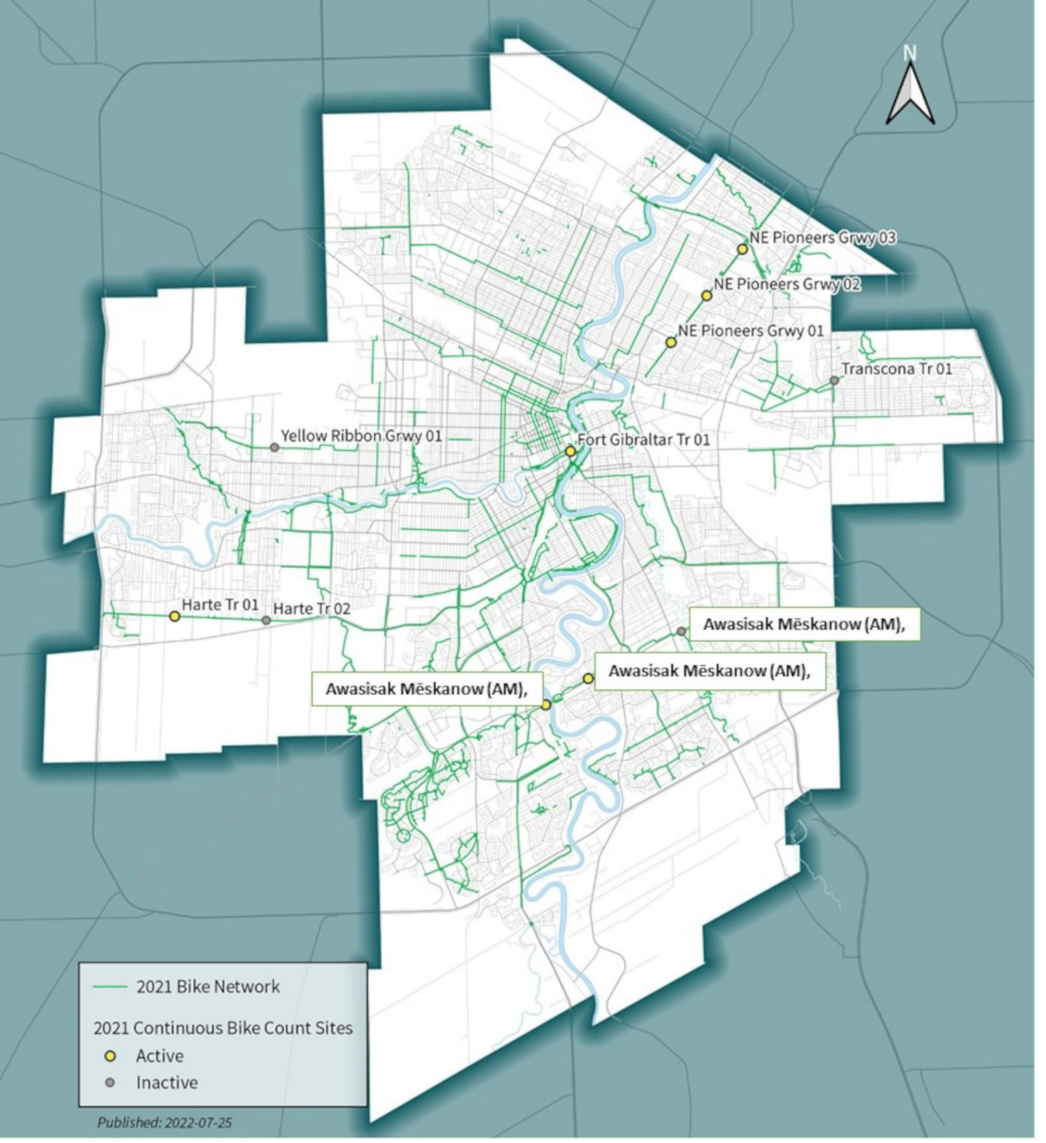
Geospatial map of the trails used to test the main study hypothesis. Green line reflect different protected cycling infrastructure in the city Circles represent the sites of various Zelt counters that quantify cycling traffic

Patterns of AT and leisure-specific cycling behaviours were quantified using open access cycling traffic data collected on five multi-use urban trails from 2014 to 2019 provided to our team from the Active Transportation Department at the City of Winnipeg. In 2014, the department embedded several automatic inductive loop detectors (Zelt 2, Eco-counter, Montreal Qc) along 5 large urban trails: Awasisak Mēskanow (AM), Northeast Pioneers Greenway (NPG), Transcona Trail (TRT), Yellow Ribbon Greenway (YRG) and the Harte Trail (HRT) (Fig. 2) to quantify patterns and trends in cycling traffic. Details for the multi-use trails on which cycling count data were collected and the population living within 400 m of the trails are provided in Table 1. Each urban trail is at least 4 km in length. There are ~ 52,000 individuals living within ~ 100 neighbourhoods within 400 m of the five trails.

**Table 1:** Neighbourhood characteristics within 400 m of each trail

	Trail A AM	Trail B NPG	Trail C YRG	Trail D TT	Trail E Harte Trail
***Trail type***	AT	AT	AT	Leisure	Leisure
***Demographics***					
Dissemination Areas (DA)	60	80	32	35	14
N	26,791	31,924	12,361	12,710	1607
Female (%)	53.26%	53.12%	52.80%	51.43%	51.8%
Age (years)	53.00	54.00	52.95	50.81	54.20
Visible Minority (%)	15.60%	10.82%	6.88%	6.37%	16.95%
Immigrated last 10 years (%)	4.95%	3.97%	2.47%	1.80%	7.8%
***Socioeconomic indicators***					
SEFI	-0.6240	-0.2165	-0.3670	-0.3108	-0.3959
Household income	$78,903.78	$67,918.18	$64,027.56	$68,255.49	$96,205
Average Property Value	$150,949.41	$116,183.20	$104,129.83	$106,676.21	$720,559
Population without high school graduation (%)	19.57%	25.39%	19.30%	24.40%	
Unemployment Rate	5.15%	4.79%	4.22%	4.97%	5.85%
***Activity indicators***					
Fitness/Recreation Centres within 5 km (2018 only)	96.20	100%	61.82%	100%	95.5%
Average Distance to greenspace (m)	192.65	154.89	162.92	160.99	125.22
Walkability Score	-0.0798	0.4680	0.0726	-0.0366	0.236
Active commuting (%)	6.25%	5.03%	5.59%	3.47%	3.94%

AT = active transportation; SEFI = socioeconomic factor index. AM = Awasisak Meskanow; NPG = Northeast Pioneers Greenway; YRG = Yellow Ribbon Greenway; TT = Transcona Trail

#### Quantification of “real world” population-based trends in active transportation and leisure cycling

**Fig. 3:**
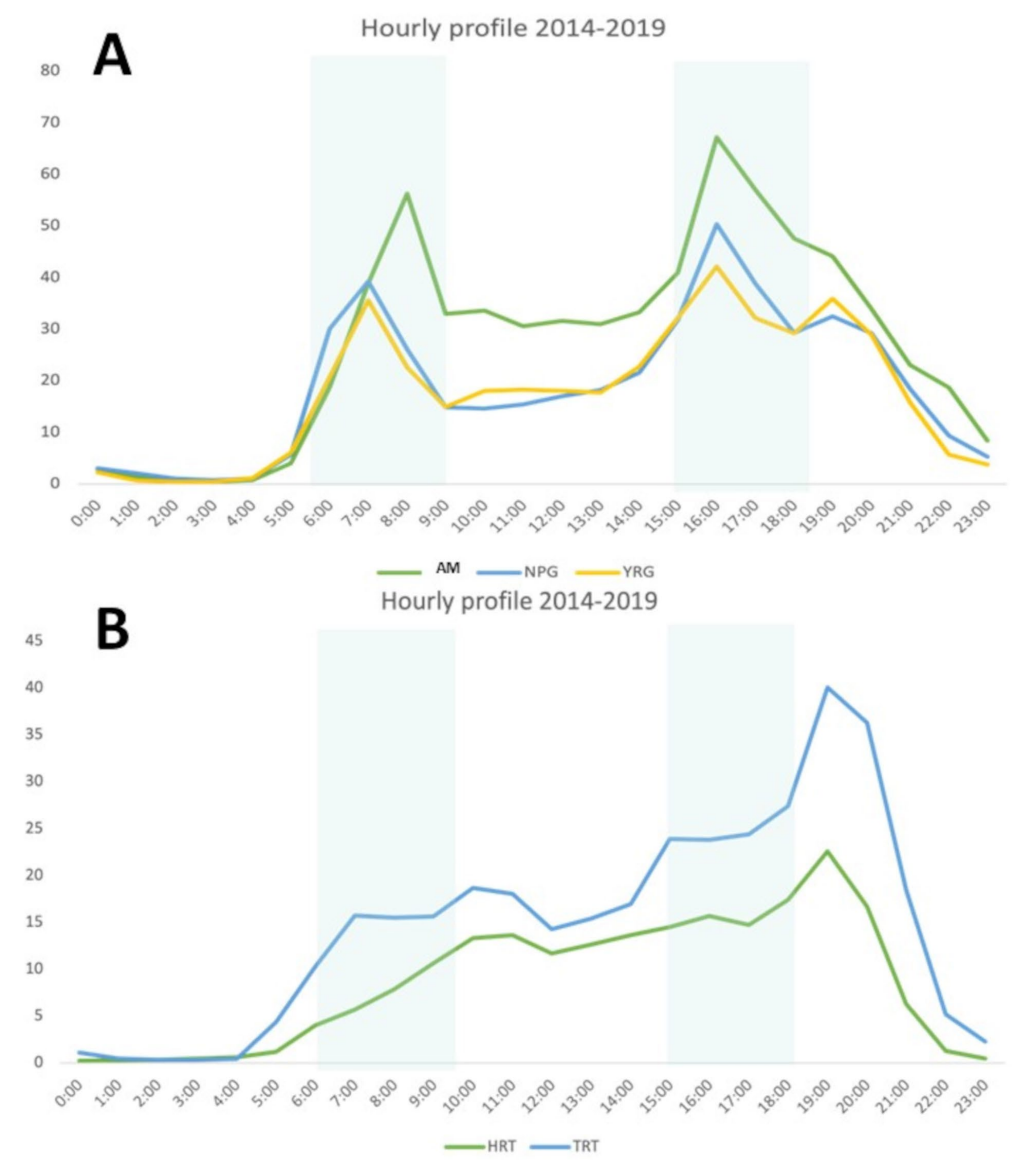
Hourly profiles of cycling traffic along the five urban trails used for the study. **(A)** Trails defined as “active transportation” based on a daily bi-phasic pattern of traffic. **(B)** Trails defined as “leisure” trails characterized by a single peak in traffic that occurred in the evening. AM = Awasisak Meskanow; NPG = Northeast Pioneers Greenway; YRG = Yellow Ribbon Greenway; HRT = Harte Trail; TRT = Transcona Trail

The automatic inductive loop detectors embedded in all five trails quantify individual bicycle counts from electromagnetic signature of the two wheels [8, 17]registering an individual count with an accuracy between 90 and 95% [18], and data are recorded and logged hourly. Hourly cycling counts were collected every day of the year from 2014 to 2019, however we restricted analyses to weeks between May 1st to September 30th as cycling rates decline by ~ 90% during winter months in Winnipeg [8]. To classify trails as “AT” or “leisure” with regards to cycling traffic patterns, we stratified hourly cycling counts into windows of active transportation (6h00-9h00 and 15h00-18h00) and leisure time cycling (9h00 to 15h00 and 18h00 to 22h00). Trails that displayed a distinct bi-phasic increase in cycling counts during the 6h00-9h00 and 15h00-18h00 windows were classified as AT trails. Trails without the bi-phasic increase in cycling counts during active transportation windows of time were classified as leisure or primarily recreational (Fig. 3). To determine if the Fresh Start Effect was evident within daily cycling patterns, we compared daily cycling counts from Monday through Friday on both AT and leisure trails. To increase the resolution for active transportation-based cycling pattern, we also conducted sensitivity analyses restricted to counts restricted to the AT windows of time.

#### Research Question #2: How do daily cycling traffic trends observed on AT trails compare to actual cycling-specific AT?

The two main limitations of the population-based real-world data used to answer research question #1 are: (1) information on origin and destination of each cycling trip were unavailable and (2) data for individual cyclists were not available, therefore it was unclear if the hourly patterns in trail use observed on the trails were for individual-level AT. To overcome these limitations, we obtained counts for daily use of a protected bicycle parking space located at Manitoba Hydro, the largest provider of electricity in the province and one of the largest employers in the City of Winnipeg with a staff of 6,463 employees. The head offices for the corporation are in downtown Winnipeg and have offered secure bicycle parking for all employees since 2012. An employee-driven AT committee provided access to a de-identified database for daily bicycle parking rates for employees that cycled to work and used the secure facility since 2012. Using this database, we tested for a Fresh Start Effect pattern of active transportation by comparing daily counts for individual use of the secured bicycle parking space from Tuesday to Friday, with data restricted to counts between May 1st to September 30th. An advantage of using this data is that the temporal landmark for these trends was Tuesday, rather than Monday. A corporate policy/benefit called “Hydro Mondays” is a 9-day bi-weekly work schedule which provides most employees with a non-work day every second Monday. These data therefore provide additional information to support the concept that the temporal landmark of interest for trends in cycling-specific AT is the start of a work week.

#### Research Question #3: How do weekly patterns of cycling behaviours compare to the original description of the fresh start effect?

Research questions #1 and #2 were designed to detect a Fresh Start Effect for cycling-specific behaviours. The original experiment that described the weekly Fresh Start Effect relied on data for individual access to a university-based fitness centre, comparing rates of attendance from Monday to Friday [12]. To replicate the original Fresh Start Effect, validate the notion that the beginning of a work week is a temporal landmark for PA behaviours and to compare the effect sizes we observed in daily patterns of AT-based cycling behaviour, we secured individual swipe card access to two fitness centres at the University of Manitoba from 2017 to 2019. To gain access to each fitness centre, attendants must swipe their access card, and the fitness centre records individual identification numbers, as well as the date and time of each attendance. The University of Manitoba supports 9421 staff/faculty and 31,020 students. Fitness centre access is provided free of charge to students and at discounted rates to staff and faculty. To answer research question #3 and compare the effect sizes observed in questions #1 and 2, we calculated absolute and relative decline in daily attendance rates at the fitness centres from Monday to Friday between 2017 and 2019.

#### Research question #4: Could daily trends in active transportation be explained by trends in office-based occupational attendance?

It is possible that the daily trends in AT-based cycling traffic over the course of a work week were related to trends in working from home or reduced work weeks during summer months. Although we were unable to obtain data for employee attendance rates in large corporate organizations, we did obtain data for vehicle traffic along one of the city’s busiest roads from May 1 to September 30th 2019. Data for other years or other roads were not available prior to 2020. Vehicular traffic in both north and southbound directions were counted using dual side-fire radar technology (Speedlane Pro, Houston Radar Inc, Sugar Land, TX). In addition to vehicle traffic, we also obtained data from coffee sales at a local provider within downtown Winnipeg, the city’s primary business sector. Parlour Coffee provided their daily sales of all types of coffee from 2014 to 2019. Parlour coffee was open Mondays through Saturdays from 7am to 3pm and is not open in the evenings. The validity of daily coffee sales as a surrogate of weekly work/occupational attendance was based on two assumptions. First, Parlour coffee serves largely a business and student clientele which is reinforced by the hours of operation and location. Second, individuals often purchase coffee prior to or during their workday. Similar to questions 1 to 3, we compared daily coffee sales and vehicular traffic from Monday to Friday to determine if sales and traffic declined on Fridays due to fewer individuals travelling to work.

### Statistical analyses

After classifying trail type, descriptive statistics were used to compare daily trends in each year that data we available for all four datasets. For each dataset, unadjusted analysis of variance (ANOVA) was used to compare daily counts across all five days of the week without adjusting for co-variates. Finally, linear mixed effect regression models with repeated measures, was used to compare daily counts between the beginning of the work week (control day) to the other days of the week, controlling for year of measurement. To test the main hypothesis (research question #1), the model included an interaction term of trail type and day of the week, to determine if the daily trends over the week were different between AT and leisure type trails. A significant interaction between trail type and day of the week would reflect differences in trends in cycling behaviour over the course of the week. Data are presented as counts with confidence intervals, and differences in counts between Mondays and other days of with week with 95% confidence intervals. Differences in daily counts were considered significant if the 95% confidence intervals do not include zero. Data for weather (obtained from federal open source data for the city of Winnipeg) and holidays were included and adjusted for in all analyses of daily cycling patterns. All analyses were conducted in RStudio 4.3.0. R code for analyses is provided in the appendix.

## Results

### Weekly trends in cycling-based AT are characterized by a fresh start effect behavioural pattern

Of the five urban multi-use trails studied, three displayed distinct daily AT profiles with bi-phasic peaks during the windows of time when AT is more common [19] (Fig. 3A) while two displayed a leisure-type cycling profile (Fig. 3B). In addition to a distinct hourly AT profile, these trails also had more cycling traffic than trails defined as leisure-type (910 270 total bicycle counts vs. 313 632 total bicycle counts from 2014 to 2019). Between 2014 and 2019, weekly cycling traffic remained similar on all five trails (eFigure 1). Trails with a distinct AT profile were built in neighbourhoods with higher population density, greater trail connectivity and more destination points, compared to trails with a leisure-type profile (Table 1).

**Fig. 4:**
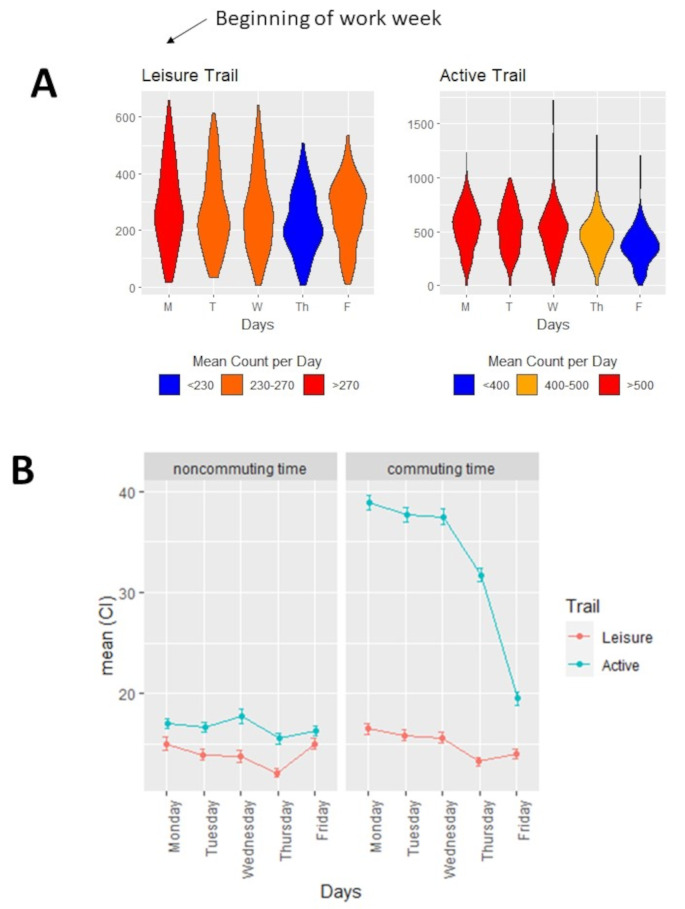
Weekly patterns in cycling behaviour along active transportation and leisure-type trails. **(A)** Weekly trends in cycling active transportation and leisure type trails **(B)** Weekly trends in cycling traffic during commuting and non commuting windows of time. Commuting windows of time were 6h00-9h00 and 15h00-18h00 Data are presented as mean counts her hour with 95% confidence intervals

In a fully adjusted linear mixed effects model, we found that the beginning of the work week was a distinct temporal landmark for AT-based cycling, after which, daily rates of cycling traffic declined progressively to the end of a work week (Fig. 4A). Trails with an AT profile observed a 22% decline cycling traffic by Friday compared to Monday (-147 cyclists/day; 95% CI: -199.0 to -94.6 cyclists/day). Urban trails defined as leisure type, were not characterized by a decline in cycling traffic between the start and end of a work week (-22.1 cyclists/day; 95% CI: -59.1 to + 15.0 cyclists/day). To increase the resolution of these trends, we repeated comparisons of daily cycling counts restricted to windows of time characterised by AT-based cycling traffic. We found that counts per hour during AT windows declined progressively through the week, with 5 fewer cyclists per hour (95% CI: -7.75 to -2.04) on Fridays compared to Mondays (eFigure 2). These trends during commuting windows were evident on trails defined as AT and leisure, suggesting they are robust to AT-specific windows of time (Fig. 4B). The trend that the beginning of each week was a temporal landmark for AT-based cycling behaviour were consistent across all five years of data collection and across all AT trails (eFigure 3).

### Daily trends in cycling on AT trails are similar to daily trends for accessing bicycle parking at a large corporate office

**Fig. 5:**
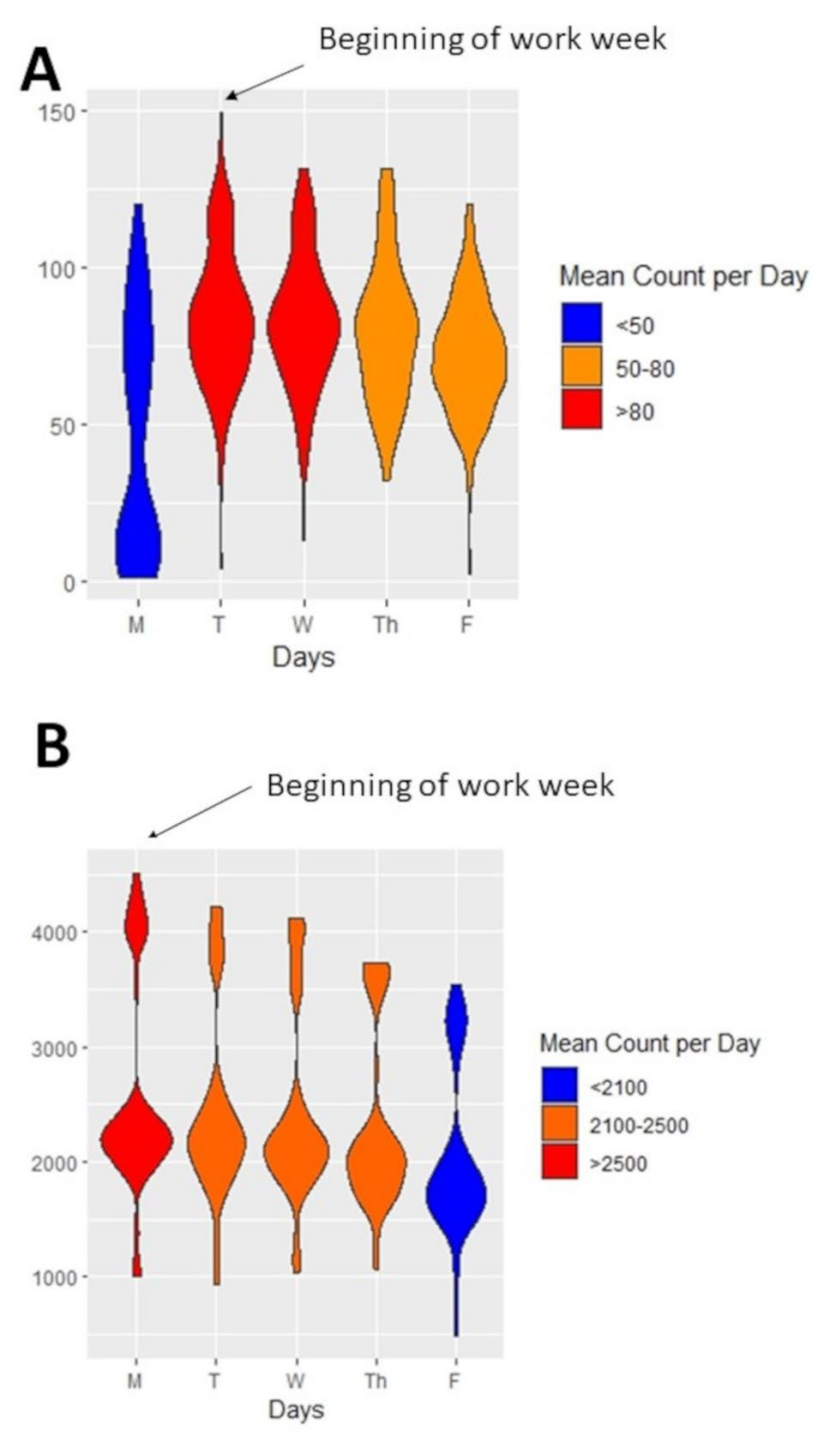
Replication of AT cycling behaviour and comparison with original fresh start effect. Weekly patterns in **(A)** Trends in parking in secure bicycle parking space at large corporation **(B)** Weekly trends in fitness centre attendance

To validate and replicate the trends observed in AT-based cycling along urban multi-use trails, we tested for differences in individual access to a protected bicycle parking space between the beginning and end of the work week. From 2012 to 2019, there were 56,307 unique accesses to the protected bicycle parking space, with annual use increasing nearly three-fold from 3640 accesses in 2012 to 9754 accesses in 2019. The temporal landmark for this dataset was Tuesday, as there is a corporate policy for a statutory holiday every other Monday for most employees of the company. Similar to the trends observed along urban multi-use trails, daily rates of bicycle parking declined by ~ 20% (-12 cyclists/day 95% CI: -17 to -7 cyclists) between Tuesday and Friday (Fig. 5a). The decline in the use of protected space for bicycle parking over a work week was evident in all 7 years of data collection (eFigure 4).

### Daily trends in cycling traffic on active transportation trails is similar to daily trends in fitness centre attendance

To determine if the magnitude of the decline AT-based cycling behaviours over the course of a work week was similar relative decline in PA described in the original Fresh Start effect [12]we examined daily trends in attendance at the local university fitness centre. Similar to the daily trends we observed for AT-based cycling traffic, visits to the fitness centre were highest on at the beginning of the work/academic week, with an average of 2,833 visits per day (95% CI: 2740–2926 visits per day) and declined progressively through the week, with the lowest visits on Fridays (Fig. 5b). The relative decline in daily visits to the fitness centres between Mondays and Fridays was 21%, with an absolute difference of -592 visits per day (95% CI: -759 to -425 visits per day). These data replicate the original findings that were used to define the Fresh Start Effect heuristic [12] and mirror the trends observed for AT-based cycling along urban multi-use trails. The decline in fitness centre attendance from the beginning to the end of the work week was evident in all three years data were available (eFigure 5).

### Trends in AT do not appear to be driven by daily patterns in working from home or four-day work weeks

**Fig. 6:**
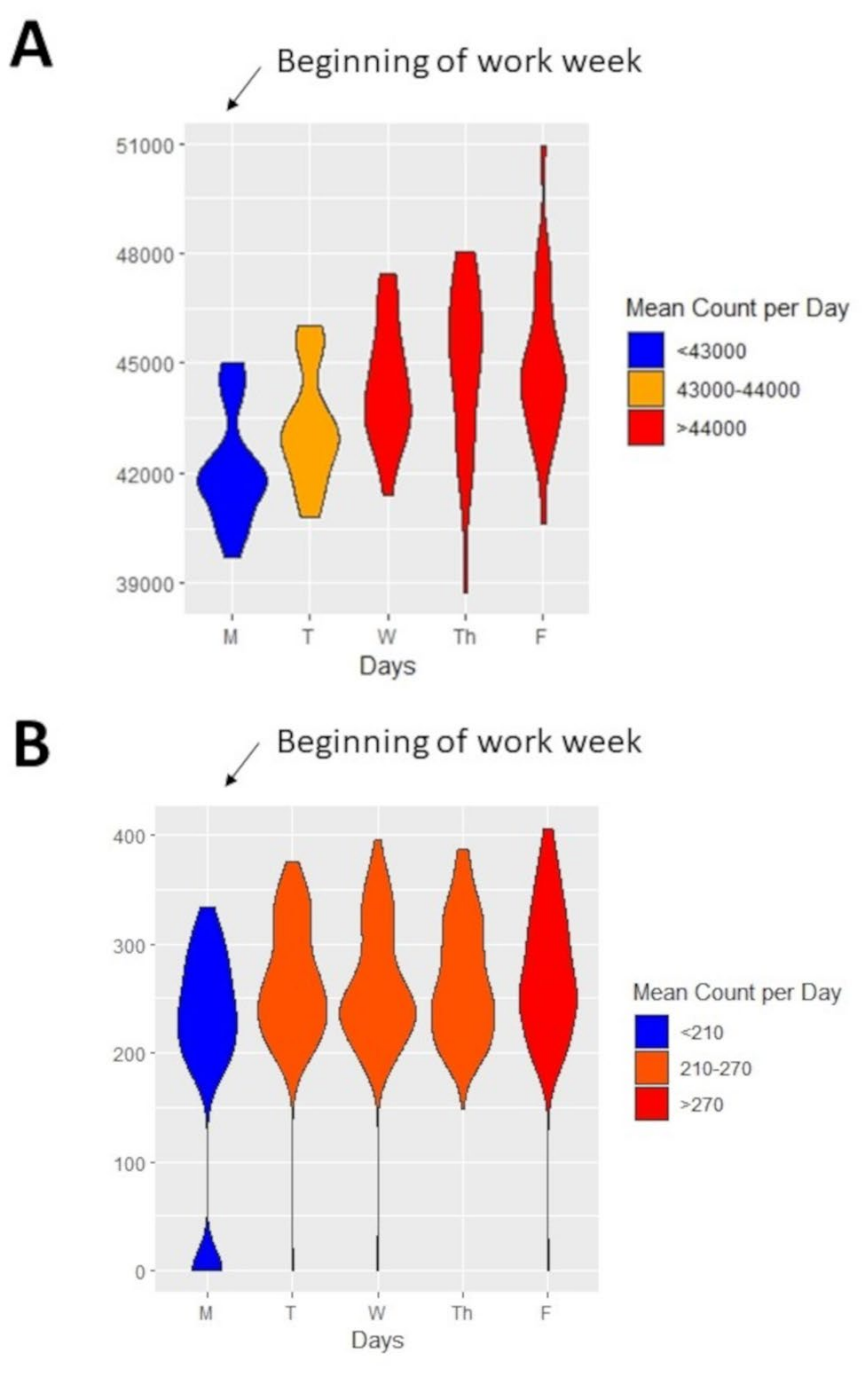
Weekly trends in negative controls for occupational attendance. Weekly patterns in **(A)** Trends in vehicular traffic along one of the cities busiest roads. **(B)** Weekly trends in coffee sales at a local coffee shop

In contrast to the trends observed in AT-based cycling behaviour along urban multi-use trails and corporate bicycle parking spaces, vehicular traffic increased 7% (+ 2848 cars/day; 95% CI: +2202 to + 3674 cars/day) from the beginning of a work week to the end of the work week (Fig. 6a). Similar to vehicular traffic patterns, coffee sales were lowest at the beginning of a work week (245 units per day; 95% CI: 234–253 units per day) and increased incrementally through the work week to a peak of 275 (95% CI: 266–286) units of coffee sold Fridays (Fig. 6b). The relative increase in coffee sales (~ 10%; 31.9 units sales per day; 95% CI: 22.0-41.7 unit sales per day) over the course of a work week was similar to the increase in vehicular traffic and was evident in all 5 years that data were available. Collectively these data imply that individuals were not working from home at the end of a work week.

## Discussion

Using real-world population-level data, we find that AT-based cycling behaviour in a large city displays a distinct weekly pattern indicative of a Fresh Start effect. Specifically, we find that the beginning of a work week is a temporal landmark for AT-based cycling behaviour that declines approximately 20% over the course of a work week. These weekly trends in AT-based cycling are particularly robust for cycling traffic between the hours of 6h00 and 9h00 and 15h00 and 18h00. This decline in cycling traffic by the end of a work week is mirrored by an increase in vehicular traffic and coffee sales at a local coffee parlour. The relative decline in AT-based cycling was similar to the relative decline in fitness centre attendance over the course of a work week. Taken together, the trends observed across all five datasets infer that AT-based cycling patterns follow a distinct Fresh Start Effect heuristic, with the beginning of the work week acting as a temporal landmark.

The promotion of AT is a growing public health strategy for reducing carbon emissions and the burden of chronic diseases in large urban centres within high income countries [20, 21]. The proportion of urban residents that engage in AT varies considerably within and between cities [8, 19, 22–27]. This variability in AT has been attributed to availability of infrastructure, localized mix land use, perceived safety and local culture for cycling [28, 29]. The data presented here add a novel behavioural driver of AT-based cycling behaviour. Specifically, AT-based cycling behaviours are sensitive to the temporal landmark of the beginning of each week. Similar to fitness centre attendance and interest in health behaviour change [12] population-level AT cycling behaviours decline progressively following a temporal landmark (beginning of a work week). At a population level, the observed 20% decline in AT-based cycling over the course of a week equates to ~ 10,000–50,000 fewer individuals travelling by bicycle in cities with populations of 1 to 5 M residents. Understanding this trend in AT-based cycling behaviours provides a novel lever for urban public health officials to nudge active commuters to sustain behaviours they adopted early in the week. For example, public health campaigns could leverage temporal landmarks to launch new initiatives to foster AT or cycling generally on Monday or Tuesdays. Officials could also leverage the trends over the work week to encourage populations to be conscious of the Fresh Start Effect and try to “flatten the curve” by continuing to engage in AT-based cycling behaviours towards the end of each week. Interestingly, we did not observe the same trend with leisure-based cycling along all trails as some trails are destination trails that are commonly used on weekend days (appendix eFigure 3). Future research using individual-level cycling data (i.e. available from Strava or Fitbit) could address these differences in daily patterns of AT and leisure-type cycling.

Large volumes of population-based behavioural data are becoming publicly available to researchers and public health officials [30]. Using these large datasets organizations can detect, track and experiment with approaches to change the behaviours of large segments of a population. As various levels of government are using principles of behavioural economics to guide policy decisions to improve the health and well-being of its citizens [31, 32]real world data can help track the effectiveness of their campaigns/policies [33]. As cities are generating and sharing large amounts of population-level data, the opportunities for urban policy makers to use these data to study and apply principles of behavioural economics are growing. This study provides a template for researchers and policy makers for how to use publicly available data to monitor trends or test hypotheses about AT or leisure-type cycling behaviours. Cities and researchers could use publicly available data to monitor the effectiveness of policies, public health campaigns or large infrastructure projects. The availability of this data could foster greater collaborations between public health officials, city planners and researchers in the area of AT and population health.

The study is strengthened by a large effect size observed at the population-level using real world, objectively-measured, trends in cycling patterns along urban trails that were consistently replicated over multiple years. Additionally, triangulating observations from real-world cycling patterns with data collected from other sources, with different limitations, enhances the interpretation of our findings. Despite these strengths, this study has several limitations. First, the data presented are descriptive in nature and we cannot conclude that there is a causal association between temporal landmarks and AT behaviours. Second, individual-level cycling data were not available to test the study hypothesis. Therefore, the weekly trends described here cannot be attributed directly to individual behaviours, rather we infer that similar trends would be observed if individual data were available. For example, although coffee sales and traffic volumes increased towards the end of the week in the datasets we had access to, they are not directly related to the cycling data. It is possible that there are alternative explanations for the increases in coffee sales and daily vehicular traffic (i.e. driving for groceries, errands and picking up a coffee while doing so), other than a shift away from AT. Third, we were able to control for several factors including yearly trends, weather patterns and holiday days, however, several co-variates were not measured including origin and destination points, sex, gender, race, ethnicity and age. Data were also collected in a medium sized urban setting in a northern climate, therefore the generalizability of findings may be limited. Specifically, the observation that the beginning of the week is a temporal landmark for AT may be limited to cities within high income countries with a typical five-day work week and protected spaces for cycling. Additionally, as demographic information was not available, it is unclear if these trends daily trends in AT were evident for individuals from different genders, ages and structurally oppressed, racialized groups. Taken together, the findings presented here require additional causal approaches to confirm the validity of these exploratory observations. Future research using data from wearable technology may be used to overcome these limitations.

## Conclusion

In a large urban city, weekly trends in AT-based cycling display a Fresh Start effect with the beginning of each work week serving as an important temporal landmark to engage in this behaviour. The trends in AT-based cycling are similar to trends used to define the original Fresh Start Effect. These behavioural patterns could be used by municipal policy makers to tailor public health messages to increase rates of AT-specific cycling behaviours.

## Electronic supplementary material

Below is the link to the electronic supplementary material.

Supplementary Material 1

## Data Availability

Geospatial data for cycling infrastructure, cycling traffic counts, vehicular traffic counts are all available within the City of Winnipeg Open Data Portal: https://data.winnipeg.ca/browse?category=Transportation+Planning+%26+Traffic+Management%26limitTo=datasets%2Cmaps%26sortBy=newest Data for corporate bicycle parking access, university of Manitoba Recreation Centre accesses and coffee sales at Parlour coffee can be made available upon request to stewards of the data.

## Abbreviations

AT: Active transportation
AM: Awasisak Mēskanow, NPG = Northeast Pioneers Greenway
TRT: Transcona Trail
YRG: Yellow Ribbon Greenway
HT: Harte Trail (HRT)
