# Supplementary material for: Urban cycling-specific active transportation behaviour is sensitive to the fresh start effect: triangulating observational evidence from real world data
Source: Int J Behav Nutr Phys Act. 2025 Jun 19;22:81. doi: 10.1186/s12966-025-01785-w (PMC12180263; doi:10.1186/s12966-025-01785-w)
Supplement: Supplementary file 1 — Supplementary Material 1 [file 12966_2025_1785_MOESM1_ESM.docx]

Fresh Start Effect and Active Transportation Appendix

eFigure 1. Year to year trends and heterogeneity of weekly cycling traffic along the five multi-use urban trails studied

eFigure 2. Hourly trail counts stratified by commuting time windows.

eFigure 3. Hourly trail counts for all five trails separated for commuting and non-commuting windows of time.

eFigure 4. Daily rates of use of a bicycle parking space from 2012 to 2019

eFigure 5. Weekly trends in fitness centre attendance for each year data were available

R code for daily urban trail cycling traffic comparisons


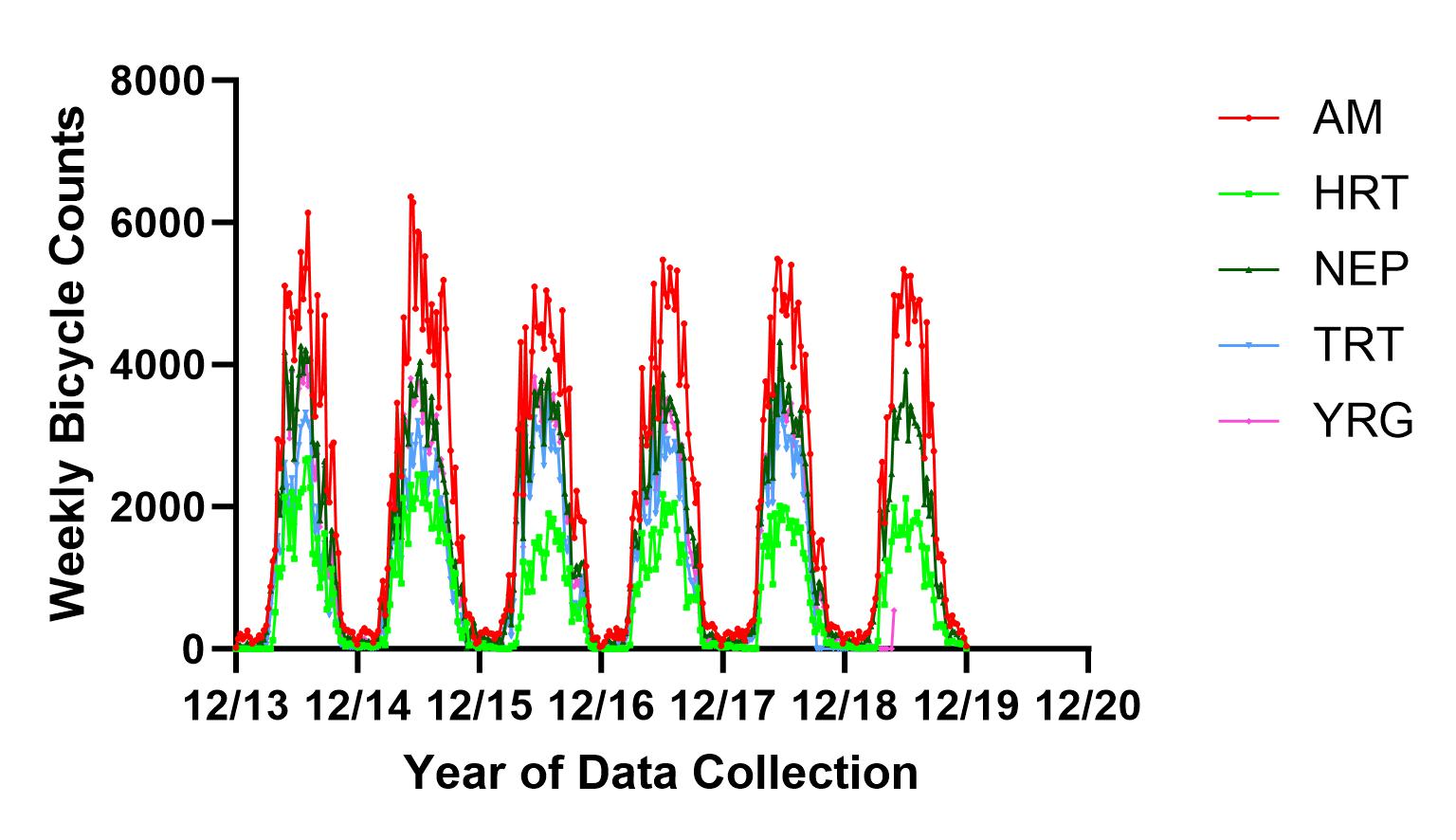


eFigure 1. Year to year trends and heterogeneity of weekly cycling traffic along the five multi-use urban trails studied

eFigure 2. Hourly trail counts during commuting hours specifically on each trail type


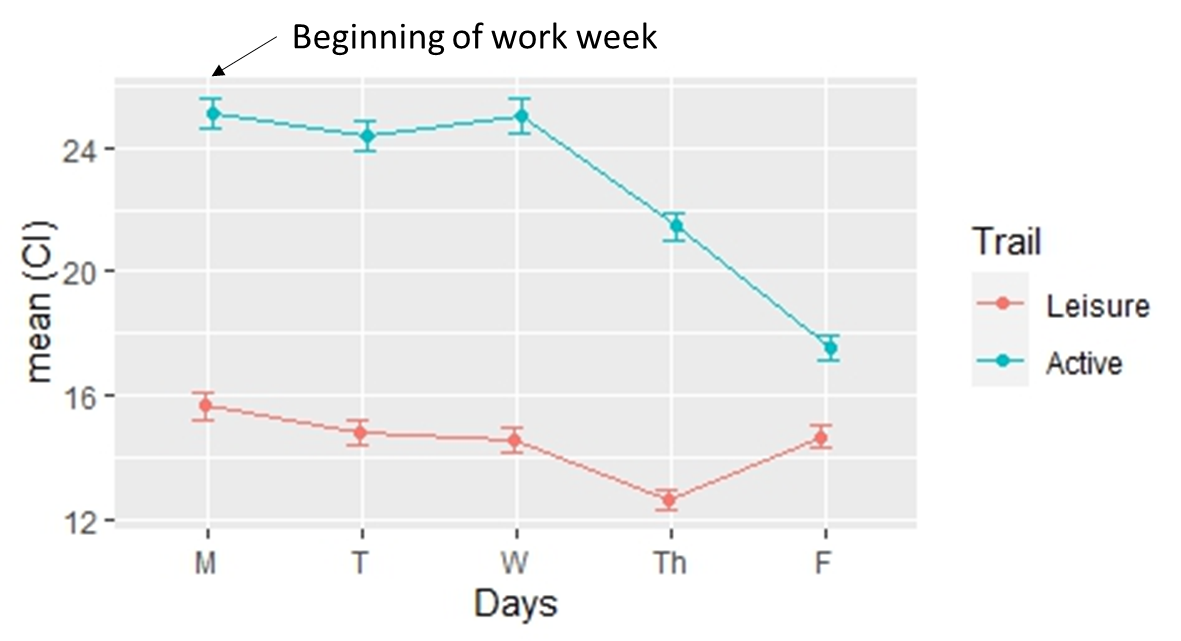


Commuting windows of time were 6h00-9h00 and 15h00-18h00

Data are presented as mean counts her hour with 95% confidence intervals


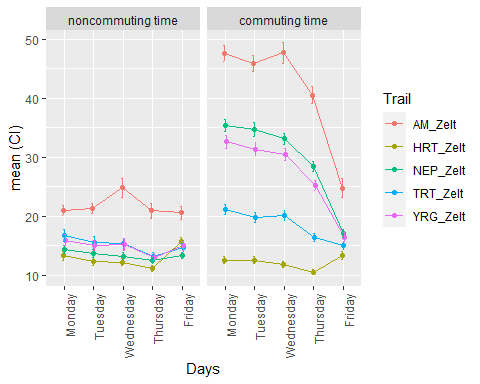


eFigure 3. Hourly trail counts for all five trails separated for commuting and non-commuting windows of time.

Noncommuting windows of time were 9h01-14h59 and 18h01-22h00

Commuting windows of time were 6h00-9h00 and 15h00-18h00

Data are presented as mean counts her hour with 95% confidence intervals

AM = Awasiak Meskanow; HRT = Harte Trail; NEP = North East Pioneers; TRT = Transscona Trail; YRG = Yellow Ribbon Trail/


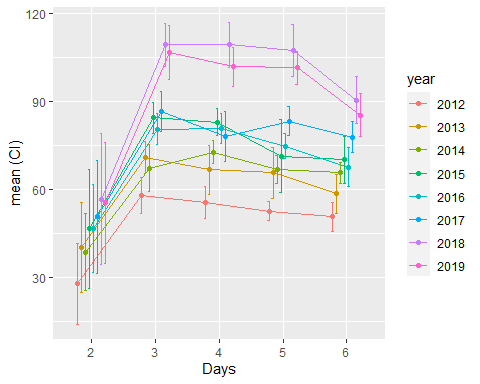


eFigure 4. Daily rates of use of a bicycle parking space from 2012 to 2019

Day 2 = Monday; 3 = Tuesday; 4 = Wednesday; 5 = Thursday; 6 = Friday.


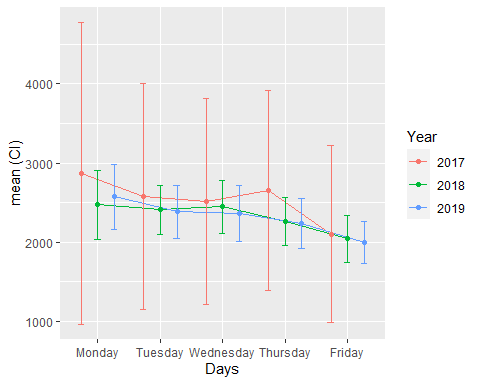


eFigure 5. Weekly trends in fitness centre attendance for each year data were available

Data are presented as a mean daily count and 95% confidence interval.

**R code for daily urban trail cycling traffic comparisons**

## Data Description and Objective

- Outcome: Daily/hourly Count of Bikes passing one of the five trails in Winnipeg
- Independent Variable: Day of the week (1: Sunday, 7: Saturday), trail type (5 types), year (2014-2020), commuting indicator (0: non-commuting time [10 am to 2 pm, 7 pm to 9 pm], 1: commuting time: [6 am to 9 am, 3 pm to 6 pm], 3: night [10 pm to 5 am])
- New commuting time variable: commuting window [6 am to 9 am, 3 pm to 6 pm] and non-commuting window [10 am to 2 pm and 7 pm to 5 am]
- Date collected hourly in between 2014 and 2020 (May-September)
- Exclusion Criteria:
  - excluded holidays and weekends
  - excluded Covid years (2020)
- There are 5 types of trails (HRT, TRT, AM, NEP, YRG), first 2 are leisure trails and rest 3 are active transportation trail.
- Objective: see the effect of time (days of the week) on count while adjusting the model for trail type, year, commuting indicator

## Data Preparation

## ── Attaching core tidyverse packages ──────────────────────── tidyverse 2.0.0 ──
## ✔ dplyr 1.1.2 ✔ readr 2.1.4
## ✔ forcats 1.0.0 ✔ stringr 1.5.0
## ✔ ggplot2 3.4.2 ✔ tibble 3.2.1
## ✔ lubridate 1.9.2 ✔ tidyr 1.3.0
## ✔ purrr 1.0.1
## ── Conflicts ────────────────────────────────────────── tidyverse_conflicts() ──
## ✖ dplyr::between() masks data.table::between()
## ✖ dplyr::filter() masks stats::filter()
## ✖ dplyr::first() masks data.table::first()
## ✖ lubridate::hour() masks data.table::hour()
## ✖ lubridate::isoweek() masks data.table::isoweek()
## ✖ dplyr::lag() masks stats::lag()
## ✖ dplyr::last() masks data.table::last()
## ✖ lubridate::mday() masks data.table::mday()
## ✖ lubridate::minute() masks data.table::minute()
## ✖ lubridate::month() masks data.table::month()
## ✖ lubridate::quarter() masks data.table::quarter()
## ✖ lubridate::second() masks data.table::second()
## ✖ purrr::transpose() masks data.table::transpose()
## ✖ lubridate::wday() masks data.table::wday()
## ✖ lubridate::week() masks data.table::week()
## ✖ lubridate::yday() masks data.table::yday()
## ✖ lubridate::year() masks data.table::year()
## ℹ Use the conflicted package (<http://conflicted.r-lib.org/>) to force all conflicts to become errors
##
## Attaching package: 'rstatix'
##
##
## The following object is masked from 'package:stats':
##
## filter
##
##
##
## Attaching package: 'Hmisc'
##
##
## The following objects are masked from 'package:dplyr':
##
## src, summarize
##
##
## The following objects are masked from 'package:base':
##
## format.pval, units

**Figures:**

working_dat%>%
 mutate(count = count %>% na_if(0)) %>%
 drop_na(count) %>%
 group_by(DayoftheWeek, trail1) %>%
 summarise(ci = list(mean_cl_normal(count) %>%
 rename(mean=y, lwr=ymin, upr=ymax))) %>%
 unnest(cols = c(ci))%>%ggplot(aes(x=DayoftheWeek, y=mean, col=trail1))+
 geom_line(aes(group=trail1), position=pd) +
 geom_point(position=pd)+ ylab("mean (CI)")+xlab("Days")+labs(col="Trail")+
 geom_errorbar(aes(ymin = lwr, ymax = upr),
 width=.3, position=pd)

## `summarise()` has grouped output by 'DayoftheWeek'. You can override using the
## `.groups` argument.

**Visuals for sensitivity analyses by commuting window time:**

working_dat%>%
 mutate(count = count %>% na_if(0)) %>%
 drop_na(count) %>%
 group_by(DayoftheWeek, trail1, CommutingBinary1) %>%
 summarise(ci = list(mean_cl_normal(count) %>%
 rename(mean=y, lwr=ymin, upr=ymax))) %>%
 unnest(cols = c(ci))%>%ggplot(aes(x=DayoftheWeek, y=mean, col=trail1))+
 geom_line(aes(group=trail1), position=pd) +
 geom_point(position=pd)+ ylab("mean (CI)")+xlab("Days")+labs(col="Trail")+
 geom_errorbar(aes(ymin = lwr, ymax = upr),
 width=.3, position=pd)+
 facet_wrap(~ CommutingBinary1) +
 theme(axis.text.x=element_text(angle=90,hjust=1))

## `summarise()` has grouped output by 'DayoftheWeek', 'trail1'. You can override
## using the `.groups` argument.

**Analyses:**

# Model Fit

# No interaction term
# For trail type, leisure trail is the reference category
# For commutingBinary, non-commuting time is the reference category
fit1 <- working_dat %>%
 mutate(count = count %>% na_if(0)) %>%
 drop_na(count) %>%
 group_by(DayoftheWeek, trail1, year, CommutingBinary1) %>%
 summarise(mean_count = mean(count)) %>%
 lm(mean_count ~ DayoftheWeek+trail1+year+CommutingBinary1, data =.)

## `summarise()` has grouped output by 'DayoftheWeek', 'trail1', 'year'. You can
## override using the `.groups` argument.

anova(fit1)

## Analysis of Variance Table
##
## Response: mean_count
## Df Sum Sq Mean Sq F value Pr(>F)
## DayoftheWeek 4 544.0 136.0 4.6408 0.001701 **
## trail1 1 3377.0 3377.0 115.2328 < 2.2e-16 ***
## year 5 93.8 18.8 0.6403 0.669440
## CommutingBinary1 1 2240.0 2240.0 76.4359 3.306e-14 ***
## Residuals 108 3165.0 29.3
## ---
## Signif. codes: 0 '***' 0.001 '**' 0.01 '*' 0.05 '.' 0.1 ' ' 1

# summary(fit1)

res1 <- as.data.frame(tidy(fit1, conf.int = TRUE, conf.level = 0.95,
 exponentiate = FALSE)%>% tbl_df %>% print(n=40))

## Warning: `tbl_df()` was deprecated in dplyr 1.0.0.
## ℹ Please use `tibble::as_tibble()` instead.
## Call `lifecycle::last_lifecycle_warnings()` to see where this warning was
## generated.

## # A tibble: 12 × 7
## term estimate std.error statistic p.value conf.low conf.high
## <chr> <dbl> <dbl> <dbl> <dbl> <dbl> <dbl>
## 1 (Intercept) 13.4 1.71 7.83 3.51e-12 10.0 16.8
## 2 DayoftheWeekTuesday -0.804 1.56 -0.514 6.08e- 1 -3.90 2.29
## 3 DayoftheWeekWednesd… -0.598 1.56 -0.382 7.03e- 1 -3.70 2.50
## 4 DayoftheWeekThursday -3.64 1.56 -2.33 2.18e- 2 -6.73 -0.538
## 5 DayoftheWeekFriday -5.56 1.56 -3.56 5.59e- 4 -8.66 -2.46
## 6 trail1Active 10.6 0.988 10.7 9.91e-19 8.65 12.6
## 7 year2015 -0.320 1.71 -0.187 8.52e- 1 -3.71 3.07
## 8 year2016 -1.20 1.71 -0.698 4.86e- 1 -4.59 2.20
## 9 year2017 -2.23 1.71 -1.30 1.95e- 1 -5.62 1.16
## 10 year2018 -1.82 1.71 -1.06 2.90e- 1 -5.21 1.57
## 11 year2019 -2.24 1.71 -1.31 1.93e- 1 -5.64 1.15
## 12 CommutingBinary1com… 8.64 0.988 8.74 3.31e-14 6.68 10.6

names(res1)[1] <- "coef"

levels1 <- lapply(names(fit1$xlevels),
 function(x) data.frame(term = x,
 coef = paste0(x, fit1$xlevels[[x]]),
 stringsAsFactors = FALSE)) %>%rbindlist()

fit.table1 <- merge(res1, levels1, all = TRUE)

fit.table1$term1 <- as.character(c("", "Commuting time","Non-commuting time",
 "Friday","Monday","Thursday","Tuesday", "Wednesday",
 "Active","Leisure","2014",
 "2015", "2016", "2017", "2018", "2019"))

fit.table1<- fit.table1 %>%
 slice(c(1,5,7,8,6,4,10,9,11,12,13,14,15,16,3,2))

fit.table1$term1 <- factor(fit.table1$term1, levels=unique(fit.table1$term1))

pd <- position_dodge(0.1)
fit.table1[-1,]%>%
 ggplot(aes(x=term1, y=estimate))+
 geom_point(position=pd)+ ylab("estimate (CI)")+
 geom_errorbar(aes(ymin = conf.low , ymax = conf.high),
 width=.3, position=pd)+xlab("")+
 theme(axis.text.x=element_text(angle = 90, hjust = 1, vjust=0.5))+
 geom_hline(yintercept = 0, lty=2)+
 geom_point(aes(x=1, y=0), pch=2)+
 geom_point(aes(x=6, y=0), pch=2)+
 geom_point(aes(x=8, y=0), pch=2)+
 geom_point(aes(x=14, y=0), pch=2)+
 labs(caption = expression(Delta ~ "is the reference category")) +
 theme(plot.caption = element_text(hjust = 0))

## Warning: Removed 4 rows containing missing values (`geom_point()`).

**Interaction term for trends by trail type:**

# interaction term (day of the week and commuting time) included
# For trail type, leisure trail is the reference category
# For commutingBinary, noncommuting time is the reference categor
# 3 Types of Interaction Model

fit3 <- working_dat %>%
 mutate(count = count %>% na_if(0)) %>%
 drop_na(count) %>%
 group_by(DayoftheWeek, trail1, year, CommutingBinary1) %>%
 summarise(mean_count = mean(count)) %>%
 lm(mean_count ~ I(DayoftheWeek:CommutingBinary1)+trail1+year, data =.)

## `summarise()` has grouped output by 'DayoftheWeek', 'trail1', 'year'. You can
## override using the `.groups` argument.

anova(fit3)

## Analysis of Variance Table
##
## Response: mean_count
## Df Sum Sq Mean Sq F value Pr(>F)
## I(DayoftheWeek:CommutingBinary1) 9 3243.0 360.3 13.8488 2.072e-14 ***
## trail1 1 3377.0 3377.0 129.7872 < 2.2e-16 ***
## year 5 93.8 18.8 0.7211 0.609
## Residuals 104 2706.0 26.0
## ---
## Signif. codes: 0 '***' 0.001 '**' 0.01 '*' 0.05 '.' 0.1 ' ' 1

# summary(fit3)

res3 <- as.data.frame(tidy(fit3, conf.int = TRUE, conf.level = 0.95,
 exponentiate = FALSE)%>% tbl_df %>% print(n=40))

## Warning: `tbl_df()` was deprecated in dplyr 1.0.0.
## ℹ Please use `tibble::as_tibble()` instead.
## Call `lifecycle::last_lifecycle_warnings()` to see where this warning was
## generated.

## # A tibble: 16 × 7
## term estimate std.error statistic p.value conf.low conf.high
## <chr> <dbl> <dbl> <dbl> <dbl> <dbl> <dbl>
## 1 (Intercept) 12.0 1.86 6.44 3.84e- 9 8.29 15.7
## 2 I(DayoftheWeek:Comm… 11.5 2.08 5.52 2.54e- 7 7.36 15.6
## 3 I(DayoftheWeek:Comm… -0.747 2.08 -0.359 7.20e- 1 -4.88 3.38
## 4 I(DayoftheWeek:Comm… 10.6 2.08 5.10 1.51e- 6 6.50 14.8
## 5 I(DayoftheWeek:Comm… -0.196 2.08 -0.0943 9.25e- 1 -4.33 3.93
## 6 I(DayoftheWeek:Comm… 10.5 2.08 5.04 2.00e- 6 6.36 14.6
## 7 I(DayoftheWeek:Comm… -2.16 2.08 -1.04 3.01e- 1 -6.29 1.97
## 8 I(DayoftheWeek:Comm… 6.38 2.08 3.06 2.78e- 3 2.25 10.5
## 9 I(DayoftheWeek:Comm… -0.369 2.08 -0.177 8.60e- 1 -4.50 3.76
## 10 I(DayoftheWeek:Comm… 0.741 2.08 0.356 7.23e- 1 -3.39 4.87
## 11 trail1Active 10.6 0.931 11.4 5.30e-20 8.76 12.5
## 12 year2015 -0.320 1.61 -0.198 8.43e- 1 -3.52 2.88
## 13 year2016 -1.20 1.61 -0.741 4.60e- 1 -4.39 2.00
## 14 year2017 -2.23 1.61 -1.38 1.70e- 1 -5.43 0.968
## 15 year2018 -1.82 1.61 -1.13 2.62e- 1 -5.02 1.38
## 16 year2019 -2.24 1.61 -1.39 1.67e- 1 -5.44 0.955

names(res3)[1] <- "coef"

levels3 <- lapply(names(fit3$xlevels),
 function(x) data.frame(term = x,
 coef = paste0(x, fit3$xlevels[[x]]),
 stringsAsFactors = FALSE)) %>%rbindlist()

fit.table3 <- merge(res3, levels3, all = TRUE)

fit.table3$term1 <- as.character(c("", "Friday:Commuting time","Friday:Non-commuting time",
 "Monday:Commuting time","Monday:Non-commuting time",
 "Thursday:Commuting time","Thursday:Non-commuting time",
 "Tuesday:Commuting time","Tuesday:Non-commuting time",
 "Wednesday:Commuting time","Wednesday:Non-commuting time",
 "Active","Leisure","2014",
 "2015", "2016",
 "2017", "2018", "2019"))

fit.table3<- fit.table3 %>%
 slice(c(1,5,4,9,8,11,10,7,6,3,2,13,12,14,15,16,17,18,19))

fit.table3$term1 <- factor(fit.table3$term1, levels=unique(fit.table3$term1))

pd <- position_dodge(0.1)
fit.table3[-1,]%>%
 ggplot(aes(x=term1, y=estimate))+
 geom_point(position=pd)+ ylab("estimate (CI)")+
 geom_errorbar(aes(ymin = conf.low , ymax = conf.high),
 width=.3, position=pd)+xlab("")+
 theme(axis.text.x=element_text(angle = 90, hjust = 1, vjust=0.5))+
 geom_hline(yintercept = 0, lty=2)+
 geom_point(aes(x=1, y=0), pch=2)+
 geom_point(aes(x=11, y=0), pch=2)+
 geom_point(aes(x=13, y=0), pch=2)+
 labs(caption = expression(Delta ~ "is the reference category")) +
 theme(plot.caption = element_text(hjust = 0))

## Warning: Removed 3 rows containing missing values (`geom_point()`).

**Adjusting for precipitation and temperature**

# interaction term (day of the week and commuting time) included with temperature and precipitation
# For trail type, leisure trail is the reference category
# For commutingBinary, noncommuting time is the reference category
fit6 <- working_dat %>%
 mutate(count = count %>% na_if(0)) %>%
 drop_na(count) %>%
 group_by(DayoftheWeek, trail1, year, CommutingBinary1, Weather, Precipitation_mm) %>%
 summarise(mean_count = mean(count)) %>%
 lm(mean_count ~ I(DayoftheWeek:CommutingBinary1)+trail1+year+Weather+Precipitation_mm, data =.)

## `summarise()` has grouped output by 'DayoftheWeek', 'trail1', 'year',
## 'CommutingBinary1', 'Weather'. You can override using the `.groups` argument.

anova(fit6)

## Analysis of Variance Table
##
## Response: mean_count
## Df Sum Sq Mean Sq F value Pr(>F)
## I(DayoftheWeek:CommutingBinary1) 9 498927 55436 452.2851 < 2.2e-16 ***
## trail1 1 382540 382540 3121.0065 < 2.2e-16 ***
## year 5 5174 1035 8.4422 5.46e-08 ***
## Weather 1 947316 947316 7728.8070 < 2.2e-16 ***
## Precipitation_mm 1 20688 20688 168.7883 < 2.2e-16 ***
## Residuals 17120 2098389 123
## ---
## Signif. codes: 0 '***' 0.001 '**' 0.01 '*' 0.05 '.' 0.1 ' ' 1

#summary(fit6)

res6 <- as.data.frame(tidy(fit6, conf.int = TRUE, conf.level = 0.95,
 exponentiate = FALSE)%>% tbl_df %>% print(n=40))

## Warning: `tbl_df()` was deprecated in dplyr 1.0.0.
## ℹ Please use `tibble::as_tibble()` instead.
## Call `lifecycle::last_lifecycle_warnings()` to see where this warning was
## generated.

## # A tibble: 18 × 7
## term estimate std.error statistic p.value conf.low conf.high
## <chr> <dbl> <dbl> <dbl> <dbl> <dbl> <dbl>
## 1 (Intercept) -8.09 0.391 -20.7 1.00e- 93 -8.86 -7.32
## 2 I(DayoftheWeek:Com… 11.8 0.389 30.2 8.65e-196 11.0 12.5
## 3 I(DayoftheWeek:Com… -0.173 0.354 -0.489 6.25e- 1 -0.868 0.521
## 4 I(DayoftheWeek:Com… 11.8 0.383 30.9 2.94e-204 11.1 12.6
## 5 I(DayoftheWeek:Com… -0.634 0.351 -1.80 7.12e- 2 -1.32 0.0548
## 6 I(DayoftheWeek:Com… 10.7 0.384 27.8 9.40e-167 9.94 11.4
## 7 I(DayoftheWeek:Com… -2.37 0.352 -6.75 1.49e- 11 -3.06 -1.69
## 8 I(DayoftheWeek:Com… 6.49 0.384 16.9 1.30e- 63 5.74 7.24
## 9 I(DayoftheWeek:Com… -1.30 0.354 -3.68 2.38e- 4 -1.99 -0.607
## 10 I(DayoftheWeek:Com… 0.442 0.387 1.14 2.53e- 1 -0.315 1.20
## 11 trail1Active 10.1 0.170 59.4 0 9.75 10.4
## 12 year2015 -1.49 0.295 -5.06 4.31e- 7 -2.07 -0.913
## 13 year2016 -1.55 0.299 -5.18 2.28e- 7 -2.13 -0.960
## 14 year2017 -2.11 0.299 -7.04 1.99e- 12 -2.69 -1.52
## 15 year2018 -3.03 0.293 -10.3 5.35e- 25 -3.60 -2.45
## 16 year2019 -2.48 0.299 -8.31 1.04e- 16 -3.07 -1.90
## 17 Weather 1.11 0.0127 87.8 0 1.09 1.14
## 18 Precipitation_mm -0.876 0.0674 -13.0 2.07e- 38 -1.01 -0.744

names(res6)[1] <- "coef"

levels6 <- lapply(names(fit6$xlevels),
 function(x) data.frame(term = x,
 coef = paste0(x, fit6$xlevels[[x]]),
 stringsAsFactors = FALSE)) %>%rbindlist()

fit.table6 <- merge(res6, levels6, all = TRUE)
fit.table6$term1 <- as.character(c("", "Friday:Commuting time","Friday:Non-commuting time",
 "Monday:Commuting Time","Monday:Non-commuting time",
 "Thursday:Commuting Time","Thursday:Non-commuting time",
 "Tuesday:Commuting time","Tuesday:Non-commuting time",
 "Wednesday:Commuting time","Wednesday:Non-commuting time",
 "Precipitation (mm)",
 "Active","Leisure","Temparature",
 "2014",
 "2015", "2016", "2017",
 "2018", "2019"))

fit.table6<- fit.table6 %>%
 slice(c(1,5,4,9,8,11,10,7,6,3,2,14,13,16,17,18,19,20,21,12,15))

fit.table6$term1 <- factor(fit.table6$term1, levels=unique(fit.table6$term1))

pd <- position_dodge(0.1)
fit.table6[-1,]%>%
 ggplot(aes(x=term1, y=estimate))+
 geom_point(position=pd)+ ylab("estimate (CI)")+
 geom_errorbar(aes(ymin = conf.low , ymax = conf.high),
 width=.3, position=pd)+xlab("")+
 theme(axis.text.x=element_text(angle = 90, hjust = 1, vjust=0.5))+
 geom_hline(yintercept = 0, lty=2)+
 geom_point(aes(x=1, y=0), pch=2)+
 geom_point(aes(x=11, y=0), pch=2)+
 geom_point(aes(x=13, y=0), pch=2)+
 labs(caption = expression(Delta ~ "is the reference category")) +
 theme(plot.caption = element_text(hjust = 0))

## Warning: Removed 3 rows containing missing values (`geom_point()`).
